# The DNA Alkylguanine DNA Alkyltransferase-2 (AGT-2) Of *Caenorhabditis Elegans* Is Involved In Meiosis And Early Development Under Physiological Conditions

**DOI:** 10.1038/s41598-019-43394-1

**Published:** 2019-05-03

**Authors:** Mario Serpe, Chiara Forenza, Adele Adamo, Noemi Russo, Giuseppe Perugino, Maria Ciaramella, Anna Valenti

**Affiliations:** Institute of Biosciences and BioResources, National Research Council of Italy, Via Pietro Castellino 111, Naples, 80131 Italy

**Keywords:** RNAi, Genetic models

## Abstract

DNA alkylguanine DNA alkyltransferases (AGTs) are evolutionary conserved proteins that repair alkylation damage in DNA, counteracting the effects of agents inducing such lesions. Over the last years AGTs have raised considerable interest for both the peculiarity of their molecular mechanism and their relevance in cancer biology. AGT knock out mice show increased tumour incidence in response to alkylating agents, and over-expression of the human AGT protein in cancer cells is frequently associated with resistance to alkylating chemotherapy. While all data available point to a function of AGT proteins in the cell response to alkylation lesions, we report for the first time that one of the two AGT paralogs of the model organism *C*. *elegans*, called AGT-2, also plays unexpected roles in meiosis and early development under physiological conditions. Our data suggest a role for AGT-2 in conversion of homologous recombination intermediates into post-strand exchange products in meiosis, and show that *agt-2* gene down-regulation, or treatment of animals with an AGT inhibitor results in increased number of germ cells that are incompatible with producing viable offspring and are eliminated by apoptosis. These results suggest possible functions for AGTs in cell processes distinct from repair of alkylating damage.

## Introduction

DNA alkylguanine DNA alkyltransferases (AGTs) are evolutionary conserved, peculiar proteins that repair alkylation damage in DNA, mainly at the O6-position of guanines, thus counteracting the effects of alkylating agents that form such adducts^1–3^. AGTs are widely present in organisms of the three living kingdoms (bacteria, archaea, eukaryotes) and typically comprise two domains: a less conserved N-terminal domain, which might play regulatory functions, and a highly conserved C-terminal domain, which houses the functional elements required for DNA binding and repair^4–10^. This latter occurs in a single-step SN_2_-type reaction, in which the alkyl moiety is covalently bound to a conserved cysteine residue in the protein active site, leading to release of an intact double strand and a protein molecule which carries the alkyl adduct^1,7,11^. This alkylated protein form is irreversibly inactivated and directed to intracellular degradation pathways^12^. AGT inactivation and degradation can also be triggered by competitive inhibitors of AGTs, such as O6-benzylguanine (O6-BG)^13^. Several lines of evidence suggest a link between AGT and cancer. First, knock out of the AGT-encoding gene in mouse results in increased tumour incidence in response to alkylating agents^14,15^. Moreover, over-expression of the human AGT protein (hAGT) in cancer cells is frequently associated with resistance to alkylating chemotherapy; for this reason, chemotherapy regimens of O6-BG in combination with alkylating agents are in clinical development^13,16^.

Over the last years, the nematode *C*. *elegans* has emerged as a model for genetic, molecular, and cellular analysis of DNA repair pathways. In particular, the *C*. *elegans* gonad is a useful toolkit for the study of germ line DNA repair as well as apoptosis, which occur both physiologically and in response to exogenous DNA damage, and their progress can be easily followed thanks to the gonad precise spatiotemporal organization^17^. Importantly, most pathways and key factors in these processes are conserved from worms to humans. These include homologous recombination (HR), non-homologous end-joining (NHEJ), mismatch repair, nucleotide excision repair, interstrand crosslinking repair^18^; orthologs of several human disease-linked genes belonging to these pathways are conserved in the nematode, including the DNA damage checkpoint gene ATR (*atl-1* in *C*. *elegans*)^19^, the breast cancer predisposition BRCA1 and BRCA2 genes (*brc-1* and *brc-2*, respectively)^20–23^, the Fanconi Anemia FANCJ, FANCD-2, FANC-I and FANCM genes (*dog-1*, *fcd-2*, *fnci-1*, *fncm-1*, respectively)^24–28^, making *C*. *elegans* a useful model to study disease-related genes.

Whereas most prokaryotic and eukaryotic species encode a single AGT ortholog, the *C*. *elegans* genome comprises two ORFs potentially coding for two distinct AGT orthologs, known as AGT-1 and AGT-2^29^. A truncated form of AGT-2 purified in recombinant form was shown to be endowed with DNA alkyltransferase activity *in vitro* and to confer resistance to alkylating agents when expressed in *Escherichia coli*^29^, thus suggesting that the protein is a *bona fide* AGT. No data about the *in vivo* function of either protein have been reported.

In this paper we used genetic tools combined with high-resolution microscopy to investigate the function of AGT-2 in *C*. *elegans*. Our data reveal that the *agt-2* gene plays unexpected roles in the nematode meiosis and early development under physiological conditions.

## Methods

### *C*. *elegans* strains and culture

All strains (Supplementary Table S1) were cultured at 20 °C under standard conditions as described by Wood^30^. The N2 Bristol strain was used as the wild type background. The *agt-2*(*tm6462*) strain was provided by the Mitani Laboratory^31^, and backcrossed three times into the N2 wild type strain. The sequence of *agt-2* wild type and *agt-2*(*tm6462*) alleles were checked by PCR amplification and sequencing using the oligonucleotides listed in Supplementary Table S2. Double and triple mutants (*agt-2; spo-11*, *agt-2; syp-2*, *agt-2; cep-1*, *agt-2; syp-2; lig-4*) were generated by genetic crosses and genotyped by PCR analysis using primers flanking each deletion. All primers are listed in Supplementary Table S2.

### RNA isolation and real-time RT-PCR

Wild type and *agt-2* young adult nematodes were broken by snap freezing in liquid nitrogen and then ground to a powder with a mortar and pestle. Total RNA was extracted using the RNeasy Plant Mini Kit (Quiagen) according to the manufacturer’s instructions. Residual DNA in RNA preparations was removed by using the DNA-free DNA Removal kit (Ambion), the absence of DNA contamination was checked by PCR analysis. Purified RNA was quantified by a Nanodrop instrument (Thermofisher) and RNA integrity was checked by 1.5% agarose gel electrophoresis. cDNA was generated using the High-Capacity cDNA Reverse Transcription Kit (Applied Biosystem) according to the manufacturer’s instructions. Reactions were performed in 50 μl and contained 5 μg of RNA, 1X enzyme mix (including Mulv and RNase inhibitor protein), and 1X RT Buffer mix (including dNTPs, random octamers, and oligo dT-16). Reactions were incubated for 60′ at 37 °C (step1) and then for 5′ at 95 °C (step 2). Real-time quantitative PCR reactions were performed using the Power SYBR Green Master Mix (Applied Biosystem), according to the manufacturer’s instructions. Each reaction was prepared in a total volume of 20 μl containing 10 ng of cDNA and 0.25 μM of primers (HPLC purified by Eurofins; sequences are listed in Supplementary Table S3). For each biological replicate, three technical replicates were run using a CFX96 Real Time System (Bio‐Rad). The RT parameters were: 36 cycles of amplification; T = 56 °C for annealing. The specificity of amplified products was checked by 1.5% agarose gel electrophoresis. The qPCR parameters were validated by the CFX Maestro software. Results were recorded as relative gene expression changes after normalizing for the housekeeping *pmp-3* gene expression and computed using the comparative CT method (2–CT) as previously described^32^. The 2–CT value was >1 for gene more highly expressed in the mutant strain; 2–CT value was <1 for gene more highly expressed in the wild-type strain. Shown are the means ± SD from three independent experiments.

### RNA interference

RNAi was performed by feeding as described previously^33^, using clones from the Ahringer library (Gurdon Institute, Cambridge, UK)^34^. The procedure is described in Supplementary Fig. S1A,B. Briefly, HT115 bacteria were transformed with a vector (L4440) for IPTG-inducible expression of double-stranded RNA (dsRNA). Animals were synchronized via standard hypochlorite treatment and grown on OP50 seeded NGM plates. L4 worms were washed with M9 buffer and transferred to fresh plates seeded with RNAi bacteria immediately and consecutively after 1 hr, 12 hrs and then every 24 hrs. Laid eggs, dead embryos and developmental defects were scored after 72 hrs.

### Screening of phenotypes

The procedure is described in the Supplementary Fig. S1B. Young adult worms were picked and individually cloned onto freshly seeded plates. Each worm was transferred to a fresh plate every 12 hrs, and laid eggs, embryonic lethality, males, developmental defects and larval arrests were scored after 72 hrs. Embryonic lethality was calculated as the ratio of the unviable eggs on laid eggs. The percentage of males, aberrant phenotypes and larval arrests was calculated as the ratio of males/aberrant/larval phenotype on hatched eggs. Data are from at least four independent experiments for each genotype.

### O6-BG treatment

The procedure is described in the Supplementary Fig. S1C. Synchronized animals were grown on OP50-seeded NGM plates in the presence of O6-BG (SIGMA) at different concentrations (0.05–2 mM). Young adult worms were picked and transferred onto fresh plates (containing the same concentration of O6-BG than the original plate) every 24 hrs for 72 hrs and embryonic lethality was calculated. The phenotypes were scored on progenies during the development. For each O6-BG concentration at least three independent experiments were performed.

### Immunolocalization

Gonads of synchronous young adult hermaphrodites were dissected, fixed, and processed for immunostaining as described^35^. The primary rabbit antibody against RAD-51^36^ was used at 1:200, The secondary antibody was the Texas Red anti-rabbit (Invitrogen) used at 1:400. The number of RAD-51 foci in each germ line zone (gonadal tip, mitotic zone, transition zone, early pachytene, mid-pachytene, late pachytene) was recorded.

### Image acquisition and analysis

Images were collected using a Leica DM6 fluorescence microscope and Hamamatsu camera under the control of Leica LAS AF 6000 software. Images were deconvolved and analyzed using the Leica LAS AF 6000 software and Image J program. Quantitative analysis of RAD-51 foci and DAPI-stained bodies along the germ line were performed on z series. Optical sections were collected at 0.18 µm and 0.50 µm increments respectively. The t-Student test for independent samples was used for the analysis of apoptosis levels and RAD-51 foci. At least five gonads were quantified for each genotype or condition.

### DNA damage sensitivity

For methylation DNA damage, synchronized L4 worms grown on standard plates were transferred to 10 ml of OP50-containing M9 Buffer (3 g/L KH_2_PO_4_, 6 g/L Na_2_HPO_4_, 5 g/L NaCl, 1 mM MgSO_4_) with or without N-methyl-N′-nitro-N-nitrosoguanidine (MNNG) or methyl-methane-sulphonate (MMS) and incubated at 20 °C with shaking. After 16 hrs worms were washed 3 times with M9 buffer and transferred to OP50 standard plates for 24 hrs. For each treatment, 9 worms were transferred to three fresh plates (three worms for each plate) and laid eggs and dead embryos were scored for 24 hrs.

For methylation treatment in combination with O6-BG, synchronized animals were grown on OP50-seeded NGM plates in the presence of O6-BG as described before. Young adult worms were picked and transferred on OP50-NGM plates containing MMS (0.5 mM) and incubated at 20 °C. After 16 hrs worms were transferred to OP50 standard plates (three worms for each plate) and laid eggs and dead embryos were scored for 72 hrs.

For camptothecin (CPT) treatment synchronized L4 worms were grown on NGM plates containing 5 μM CPT (Sigma-Aldrich) at 20 °C. The gonads were dissected immediately after CPT-treatment (T_0_) or incubated in standard conditions for 8 h (T 8) and 24 h (T 24) and analyzed by immunolocalization as described before.

### Apoptosis assay

Apoptotic germ corps were measured by Syto-12 vital staining as previously described^35^. To measure apoptosis levels after DSB induction, synchronized young adult worms were exposed to 20 Gy of γ-rays using a Caesium-137 source. Following treatment, worms were transferred to OP50-seeded plates, incubated at 20 °C and analysed after 2 hrs. To measure apoptosis levels after DNA alkylation damages, worms were treated with MMS and analysed at young adult stage. Each condition was replicated at least three times in independent experiments. Apoptotic corps from at least 80 gonads were quantified for each genotype or condition.

### Quantitative analysis of DAPI-stained bodies in diakinesis nuclei

Synchronized adult worms were transferred to glass slides in 15 μl of M9 solution. Samples were permeabilized, fixed and washed with absolute ethanol, then 15 μl of a 2 ng/μl solution of 4′, 6′-diamidino-2-phenylindole hydrochloride (DAPI) diluted in M9 was added. Quantitative analysis was performed on z series of images acquired using a Leica DM6000 fluorescence microscope, Leica DC 350 FX camera under the control of Leica LAS AF 6000 software. Optical sections were collected at 0.50 μm increments.

## Results

### The *agt-2* gene is involved embryonic and post-embryonic development

**Figure 1:**
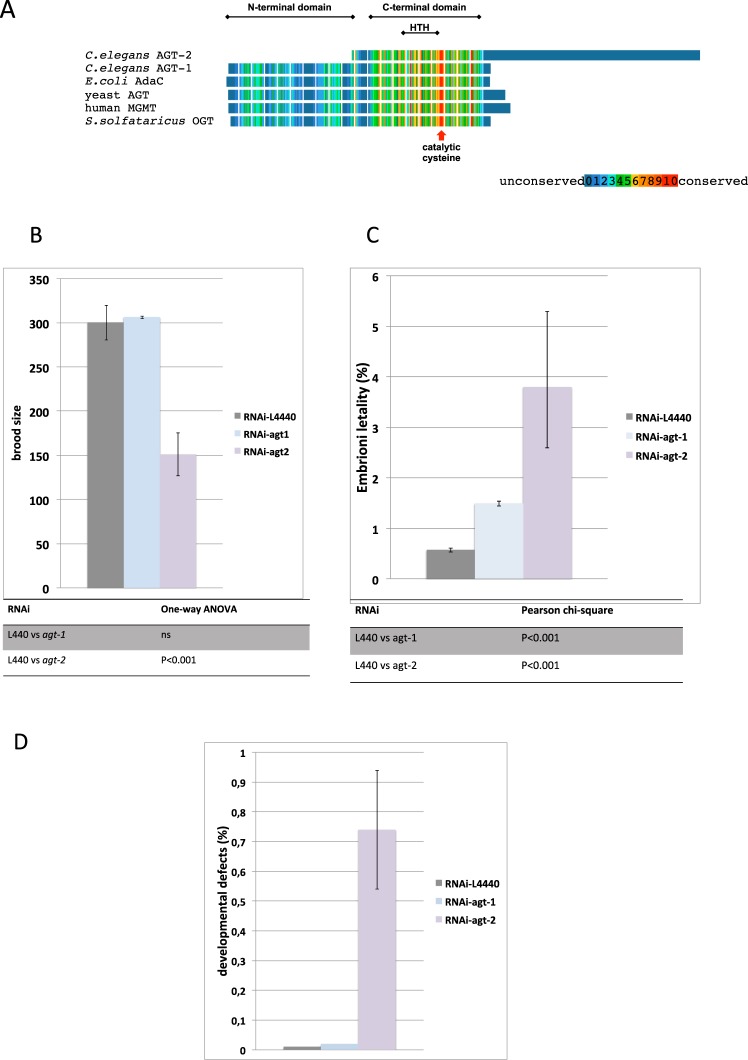
(**A**) Schematic representation of sequence alignment of selected AGTs. The N- and C- terminal AGT domains, the highly conserved catalytic cysteine and the HTH motif are indicated. The long blue C-terminal extension in AGT-2 is the domain with weak similarity with histone H1^29^. The alignment was obtained by the PRALINE program (http://www.ibi.vu.nl/programs/pralinewww/) and adjusted manually to fit the highly divergent AGT-2 sequence. (**B**–**D**) Silencing of *agt-1* and *agt-2* by RNAi. See Supplementary Fig. 1B,C for the scheme of the experiment. Histograms show: brood size (**B**); embryonic lethality, plotted as a percentage of the hatching of each genotype (**C**); developmental defects, plotted as a percentage of the hatching of each genotype (**D**). Statistical analysis is reported below each histogram (ns = not significant). Number of scored P0 worms: N2 (L440) = 27; N2 (*RNAi-agt-*1) = 27; N2 (*RNAi-agt-2*) = 36.

Of the two *C*. *elegan*s AGT paralogs, the putative AGT-1 protein (ORF Y62E10A.5) is structurally similar to other members of this protein family, whereas AGT-2 (ORF F09E5.13) predicted primary sequence is highly divergent^29^. This 274 amino acid protein displays significant similarities to canonical AGTs only in the region from residues 62 to 96, which contains the cysteine acceptor site and the DNA helix-turn-helix (HTH) binding motif; even in this region, the active site motif is –PCHP– instead of –PCHR– found in hAGT, and few of the residues in the presumptive HTH motif are identical to the corresponding residues in hAGT (Fig. 1A and Supplementary Fig. 2A). More strikingly, the N-terminal domain found in all other AGTs is totally absent in AGT-2. In contrast, AGT-2 has a much longer C-terminal sequence, not found in any known AGTs, which is rich in proline and basic amino acids and shows weak (about 25%) sequence similarity with human histone 1 C (Fig. 1A and Fig. S2A). Whereas the N-terminal portion did show alkyl-transferase activity *in vitro*, the whole protein could not be obtained in recombinant form (29 and our unpublished results); thus, the function of the AGT-2 C-terminal domain has not been established.

Given these puzzling observations, we aimed at investigating the function of the two *C*. *elegans* AGT-encoding genes. We thus down-regulated the *agt-1* and *agt-2* genes by RNAi and subjected interfered animals to phenotypic screening: whereas RNAi of *agt-1* had no apparent effect on fertility and embryonic lethality, down regulation of *agt-2* resulted in significant reduction of the brood size, and increase in the frequency of dead embryos as well as post-embryonic developmental defects (Fig. 1B–D).

This result suggests that AGT-2 plays a role in *C*. *elegans* physiology under normal growth conditions and was unexpected, since all available data point to a function of AGT proteins in repair of alkylating agents. We thus decided to investigate the role of *agt-2* in the nematode biology.

**Figure 2:**
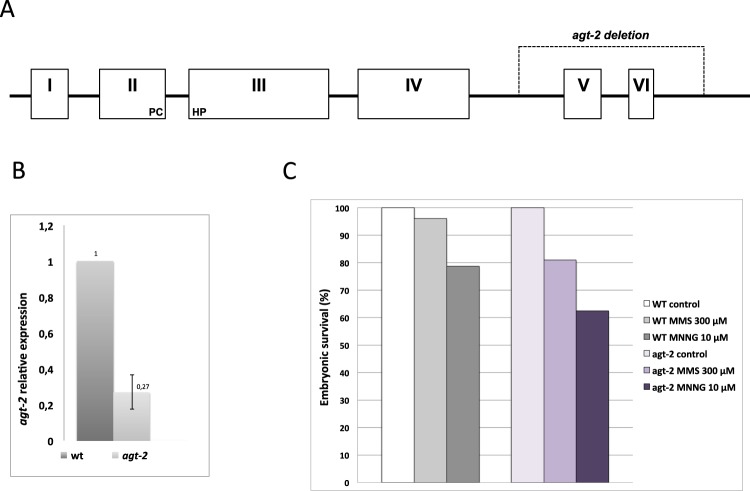
(**A**) Schematic representation of the *agt-2* gene structure. Exons are indicated by numbered boxes and introns by black lines. Residues PHCP of the catalytic site (exons II and III) are indicated. The dashed line span exons V and VI represents the portion deleted in the mutant *agt-2*(*tm6462*) allele. (**B**) Analysis of *agt-2* gene expression in the N2 background by real-time RT–PCR. Data were normalized to the expression level of the *pmp-3* housekeeping gene as previously described^35^ and expressed as the relative mRNA level compared with the average expression level in wild type animals. The mean of three independent experiments from two biological replicates is shown. (**C**) Effect of alkylation damage on wild type and *agt-2* mutant worms. L4 worms were treated with either MMS or MNNG at the indicated concentrations. Embryonic viability 48 hrs after DNA damage treatment was plotted as a percentage of the hatching normalized by that in untreated worms of each genotype. Number of scored P0 worms: untreated wild type = 9; MMS-treated wild type = 18; MNNG-treated wild type = 18; untreated *agt-2* = 18; MMS-treated *agt-2* = 18; MNNG-treated *agt-2* = 18. (chi-squared test: WT-MMS vs agt-2 MMS P < 0.001; WT-MNNG vs agt-2 MNNG P < 0.001).

In order to confirm the RNAi results we looked for stable *agt-2* mutants in *C*. *elegans* genetic banks and retrieved the *tm6462* strain, we will refer to as the *agt-2* mutant. This strain holds a 369 bp deletion spanning the last two *agt-2* exons, the last intron and part of the 3′ UTR (Fig. 2A). If translated, a full-length transcript covering the whole *agt-2* (*tm6462*) allele would eventually result in a protein identical to the wild type AGT-2 protein up to residue 234. This putative deleted protein would contain the predicted catalytic motif and, based on the results obtained *in vitro* on a shorter truncated protein^29^, could in principle catalyse the trans-alkylation reaction; however, we cannot currently make any prediction on the effect of the truncation of the C-terminal portion on the protein activity/stability. Quantitative RT-PCR analysis showed that the level of *agt-2* transcripts is reduced by about five fold in the *agt-2* mutant as compared to the wild type (Fig. 2B), thus suggesting that the *tm6462* deletion induces down-regulation or destabilization of the *agt-2* mRNA. We conclude that, even if the mutant AGT-2 version encoded by the *agt-2* (*tm6462*) allele was as functional as the wild type protein, its level would be only 20% of that found in the N2 strain. Thus, *agt-2* (*tm6462*) can be considered at least a hypomorphic mutant. A parallel analysis detected no *agt-1* transcripts in either wild type or *agt-2* strains (data not shown) suggesting that, under the conditions used, this gene is not expressed or its transcript level is below the detection threshold of the experiment.

Since the *agt-1* expression could be subjected to a trigger, we analysed the transcript after DNA damage induction, however no transcript was found after treatment of wild type or *agt-2* strains with alkylating agent MMS (data not showed).

We tested the sensitivity of the *agt-2* mutant to two monofunctional alkylating agents, namely MNNG and MMS (Fig. 2C). The *agt-2* mutant resulted reproducibly, although not dramatically, more sensitive to both drugs, as compared to the wild type (p < 0.001 for both treatment). The moderate sensitivity of the *agt-2* mutant to alkylating damage is compatible with the notion that these lesions can be repaired by multiple repair systems^37^. Thus, these results confirmed that the AGT-2 protein is involved in efficient repair of DNA alkylation lesions and that the *tm6462* deletion impairs, at least in part, this activity.

**Figure 3:**
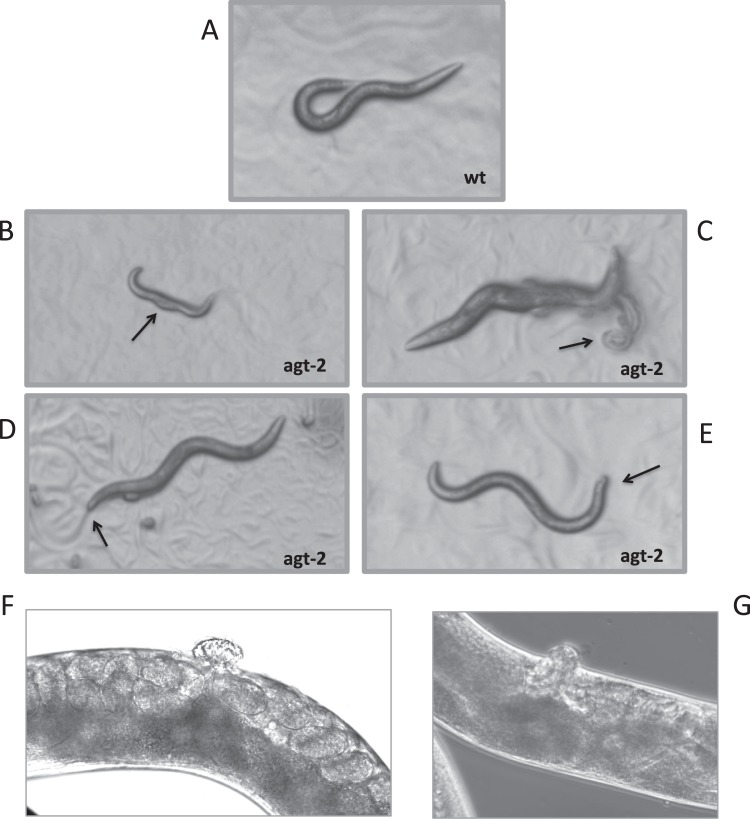
Developmental defects found in *agt-2* mutant populations. (**A**) A typical N2 worm at L4 stage. (**B**) larval arrest with a bulging of the body. (**C**) A dead worm with burst vulva. (**D**,**E**) Animals with misshapen tail. (**F**,**G**) Magnification of protruding/ectopic vulva.

In the above experiment we noticed a higher number of dead progeny in untreated *agt-2* as compared to wild type worms (not shown), as observed in the *agt-2 RNAi*. To confirm this observation, we performed systematic phenotypic screening of *agt-2* mutant worms (Table 1). Interestingly, this strain showed significant reduction of brood size and increase in embryonic lethality as compared to the wild type; in addition, we observed elevated rate of larval arrests in the *agt-2* population and, among the live progeny, of developmental defects (Table 1 and Fig. 3). In contrast, the incidence of males, reflecting the frequency of X-chromosome non-disjunction in *C*. *elegans* meiosis, was similar in the *agt-2* and wild type strains (Table 1).

**Table 1:** Screening of phenotypes in *agt-2* and wild type populations.

	*WT*	*agt-2*
Parental	20	28
Laid eggs	5341	5769
Hatched eggs	5314	5699
Brood size	267	206
Dead embryos (%)	0.5	1.2
Males (%)	0.06	0.04
Larval arrest (%)	0	0.9
Developmental defects (%)	0.04	0.8

Embryonic lethality was calculated as the percentage of unviable eggs on the total laid eggs. Developmental defects were calculated as the percentage of aberrant larval phenotypes on hatched eggs.

Collectively, these data suggest that, as expected, *agt-2* contributes to *C*. *elegans* response to alkylating agents; surprisingly, it is also involved in embryonic and post-embryonic development under normal growth conditions.

### AGT-2 protein inhibition induces embryonic lethality and developmental defects

O6-BG is a competitive and highly specific inhibitor of most, although not all AGTs, since it reacts with the active site cysteine forming a covalent adduct, which inactivates irreversibly the protein. In human cells, O6-BG induced AGT inactivation triggers the protein degradation; thus, the inhibitor is being tested to improve the efficacy of chemotherapeutic regimens based on alkylating drugs^16^. A truncated form of AGT-2 was reported to be sensitive to the inhibitor *in vitro*^29^, thus suggesting that the protein might be O6-BG target *in vivo* as well; however, the effects of O6-BG on live worms have never been tested.

We thus exposed wild type animals to different concentrations of the inhibitor and performed phenotypic screenings (Table 2). Worms treated with the drug at 0.05 mM showed brood size reduction and increased embryonic lethality as well as developmental defects, as compared to control worms; interestingly, these phenotypes were very similar to those observed in *agt-2* and *agt-2* (*RNAi*) animals. Higher concentrations of O6-BG (2 mM) exacerbated all phenotypes and in particular induced high rate of embryonic lethality and developmental defects (Table 2).

**Table 2:** Screening of phenotypes in wild type worms after treatment with O6-BG at the indicated concentrations.

	Control	O6-BG (0.05 mM)	O6-BG (2 mM)
Parental	18	18	18
Laid eggs	4046	3250	3145
Brood size	225	181	175
Dead embryos (%)	0.5	2.9	15
Developmental defects (%)	0	0.8	7

The percentage of embryonic lethality was calculated as the ratio of the total unviable eggs on the total laid eggs. The percentage of developmental defects was calculated as the ratio of aberrant larval phenotypes on hatched eggs.

We also tested the sensitivity of O6-BG treated worms to alkylation agent MMS showing an increased embryonic lethality as compared to O6-BG untreated worms.

(wt-MMS = 2.5%; wt-O6-BG-MMS = 4.2%; WT-MMS-O6-BG = 2.4%; Supplementary Fig. 2B).

Taken together the results are consistent with the prediction that the observed phenotypes are due to inactivation and/or degradation of the AGT-2 protein, reinforcing the hypothesis that the protein plays a role in DNA damage repair and also in the important step(s) of *C*. *elegans* embryonic and post-embryonic development in the absence of exogenous DNA damage.

### AGT-2 is involved in meiotic DSB repair

The above experiments clearly showed that embryonic lethality is increased when the *agt-2* gene is mutated or down-regulated, and when wild type animals are treated with an inhibitor of AGT proteins. In general, elevated levels of embryonic lethality can be associated to problems arising during *C*. *elegans* meiosis. In order to identify the nature of the *agt-2* mutant defect, we analysed the key meiotic steps in these animals. Earlier in meiotic prophase -after premeiotic DNA replication- homologous chromosomes recognize each other and pair. Chromosome pairing is coupled with the assembly of the synaptonemal complex (SC), and is followed by formation of multiple physiological double strand breaks (DSBs) induced by the SPO-11 protein, which trigger meiotic recombination (for recent review see^18^). Homologous pairing and recombination are tightly coordinated to ensure the proper chromosome inheritance.

To establish whether *agt-2* animals have defects in chromosome pairing or synapsis, we performed cytological analysis of mutant worms from the leptotene to diplotene stages of meiosis I, and we found no morphological defects that might suggest homolog alignment or SC problems (data not shown). In addition, we stained mutant and wild type gonads with an antibody against the SYP-1 protein, an SC component^38^. The SC complex appeared normal in *agt-2* mutants (Supplementary Fig. 3), thus suggesting that these latter do not have major problems in homologous chromosome pairing and synapsis.

**Figure 4:**
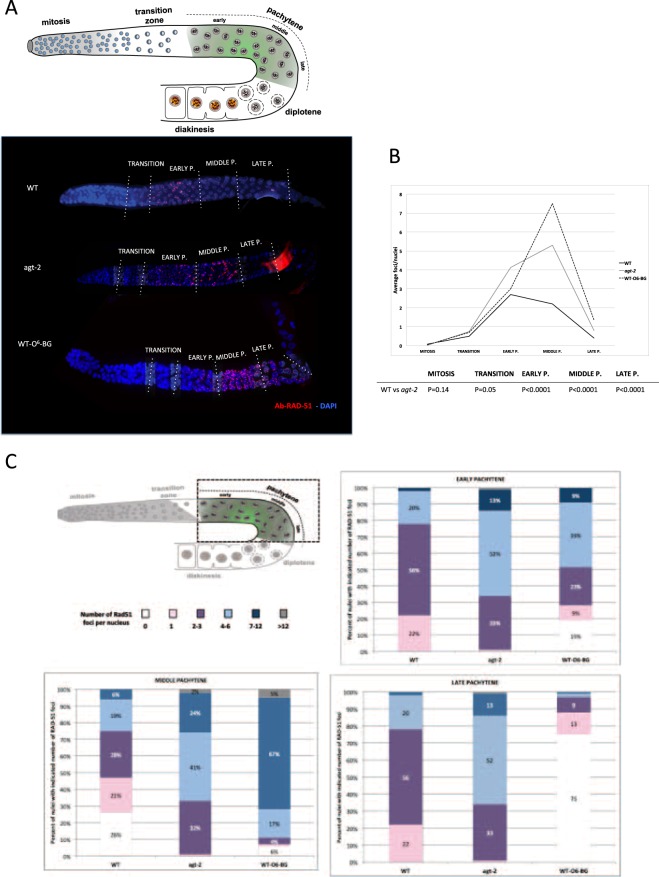
Analysis of RAD-51 foci in *C*. *elegans* germline. (**A**) Upper panel: schematic depiction of a single gonad arm. Germ cells are arranged in a “conveyor belt-like” fashion with mitotic germ cells at the distal end of the gonad arm (mitosis). As cells move along the distal arm they enter meiosis in the transition zone and progress through the different stages of pachytene. Starting from transition zone, the DBSs are formed and then repaired along the pachitene. In diplotene through diakinesis, the paired chromosomes condense to form bivalents that will be packaged into each oocyte. In the lower panel, representative images of DAPI stained DNA (blue) and RAD-51 foci (red) detected with the anti-RAD-51 antibody in the indicated zones of the gonad (P.: pachytene). (**B**,**C**) Quantitative analysis of RAD-51 foci distribution in WT, WT-O6-BG and *agt-2* animals. (**B**) The y-axis shows the average of RAD-51 foci per nucleus; the x-axis represents the position (zone) along the gonad. At least five gonads were scored for each genotype or treatment. Statistical analysis was carried out using the two-tailed paired Student’s t tests^27,35^. A significance value of p < 0.05 was used. (**C**) Upper panel: schematic drawing of pachytene indicating the position of the zones scored for RAD-51 foci. In the lower panel, stacked bar graph depicting quantitation of RAD-51 foci in early pachytene nuclei (EARLY P.), middle pachytene nuclei (MIDDLE P.) and late pachytene nuclei (LATE P.) of indicated genotypes. Different colored segments in the histograms represent the percentage of nuclei scored with the indicated number of RAD-51 foci (see the color code on the top of the graph).

We then wondered whether *agt-2* mutants initiate correctly meiotic recombination. To answer this question, we used antibodies against the DNA strand-exchange protein RAD-51^36^. RAD-51 binds single-stranded regions adjacent to resected DSBs, forming distinct foci, which follow precise kinetics in meiotic prophase and are widely used as a cytological marker of recombination intermediates and DBSs after DNA damages. Increase and/or persistence of such foci reflect a DNA repair defect^36,39^. We observed that the number of RAD-51 foci was significantly higher in *agt-2* as compared to wild type oocytes (Fig. 4A,B). In addition, quantitative analysis of RAD-51 foci in the different zones along the gonad showed that the foci persistence followed a different kinetics in the two strains: in the wild type, the number of RAD-51 foci typically increased from zygotene to early pachytene, declined in mid pachytene and disappeared by late pachytene, indicating that meiotic DSBs had been successfully repaired (Fig. 4B,C). In the mutant, the peak of RAD-51 foci was shifted to mid pachytene, and a significant number of foci persisted until late pachytene. In both strains RAD-51 foci were absent in the mitotic zone (Fig. 4B,C).

Interestingly, wild type animals treated with 2 mM O6-BG also showed increased number and persistence of RAD-51 foci as compared to controls; in this case, the number of RAD-51 foci per nucleus was even higher than in the *agt-2* mutant (Fig. 4A–C).

**Figure 5:**
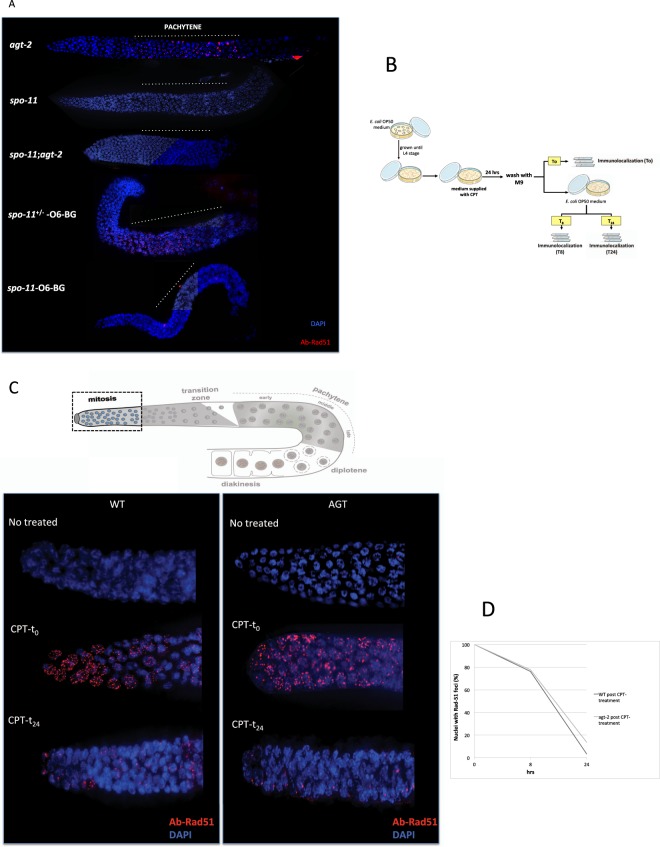
(**A**) Elevation of RAD-51 foci in the absence of a fully functional AGT-2 is dependent on meiotic DSBs. Gonads from young adult hermaphrodites were dissected and processed for immunofluorescence using the anti-RAD-51 antibody (red). DNA was visualised by DAPI staining (blue). All gonads are orientated with the distal end to the left and the proximal end to the right. Dashed lines shows the pachytene zone. Representative images of the indicated genotype or treatment are shown. At least three gonads were analyzed for each genotype or treatment. The *spo-11*+*/−* heterozygote was used as a positive control for the O6-BG treatment. (**B**–**D**) Formation of RAD-51 foci in germline nuclei in response to CPT. (**B**) Schematic representation of CPT treatment: L4-stage WT and *agt-2* mutant were treated with 5 μM CPT for 24 h. (**C**) Upper panel: Schematic depiction of *C*. *elegans* germline highlights the position of mitotic nuclei scored for RAD-51 foci. Lower panel: representative images of fixed mitotic nuclei at the distal end of the germline immunostained with RAD-51 antibody and counterstained with DAPI. The WT and *agt-2* mutant gonads were processed immediately post CPT treatment (t_0_) or after 24 h (t_24_). (**D**) The percentage of germline nuclei with RAD-51-positive foci in the gonad tip were determined at indicated times post CPT treatment.

The fact that RAD-51 foci were not observed in mitotically dividing oocytes in the *agt-2* mutant or in the presence of O6-BG suggests that the defect we are observing is meiotic and not a consequence of problems occurring during premeiotic replication. In order to confirm this hypothesis, we generated an *agt-2; spo-11* double mutant and analysed the presence of RAD-51 foci in this strain: as shown in Fig. 5A, no RAD-51 foci were observed in this mutant, as in the *spo-11* strain. In addition, when the *spo-11* mutant was treated with O6-BG, virtually no RAD-51 foci were observed, whereas, as expected, a high number of RAD-51 foci were present in meiotic prophase in the *spo*-*11*/+ heterozygous treated with O6-BG (Fig. 5A).

In conclusion, the increased number of SPO-11-dependent RAD-51 foci in meiotic prophase and their persistence indicates that a defect in repairing meiotic DSBs occurs when *agt-2* is mutated or wild type animals are treated with the AGT inhibitor. These results suggest a role for AGT-2 in the efficient repair of SPO-11-dependent meiotic DSBs.

The defects observed in *agt-2* mutant may also reflect a role of AGT-2 in sensing the presence of DNA damage. To test this possibility we checked the RAD-51 foci formation after DNA damage (DBSs) in mitotic germline nuclei. The DBSs were inducted by the topoisomerase I inhibitor camptothecin (CPT), which causes replication fork stalling and collapse in actively cycling cells (Fig. 5B-D). After treatment with CPT (CPT-t_0_) the RAD-51 foci are largely diffuses in mitotic nuclei both in wild type and *agt-2* mutant, suggesting that the accumulation of RAD-51 at damage-inducted DBSs doesn’t depends from AGT-2 (Fig. 5C,D). We also examined the kinetic of DBSs repair from 24 hrs post CPT treatment (CPT-t_24_) demonstrating that more then 80% of the nuclei did not show RAD-51 foci after 24 hrs, both in WT and *agt-2* mutant (Fig. 5C,D). Taken together these results imply that AGT acts upon spo-11 dependent DNA breaks and is dispensable for sensing and repair the DNA damage.

### AGT-2 involvement in crossover formation and inter-sister recombination

By late pachytene, homologous recombination leads to the formation of one and only one inter-homolog crossing over (CO) event per each chromosome. The rest of SPO-11 induced DSBs are repaired by non-CO mechanisms. During late prophase, SC disassembles and homologous chromosomes remain linked by chiasmata (physical attachments provided by COs in combination with sister chromatid cohesion). At diakinesis each condensed chromosome pair forms a compact body, which can easily visualized by DNA staining (e. g. DAPI); diakinetic nuclei normally contain six such bodies^18^. In contrast, if crossing over is prevented either through loss of the synaptonemal complex (SC; e. g., in *syp* mutants) or a block to meiotic DSB formation (e. g., in *spo-11* mutants), then 12 unsynapsed chromosomes (univalents) are observed at diakinesis. Thus, the morphology and number of diakinesis chromosomes are a useful readout of meiotic recombination defects.

**Figure 6:**
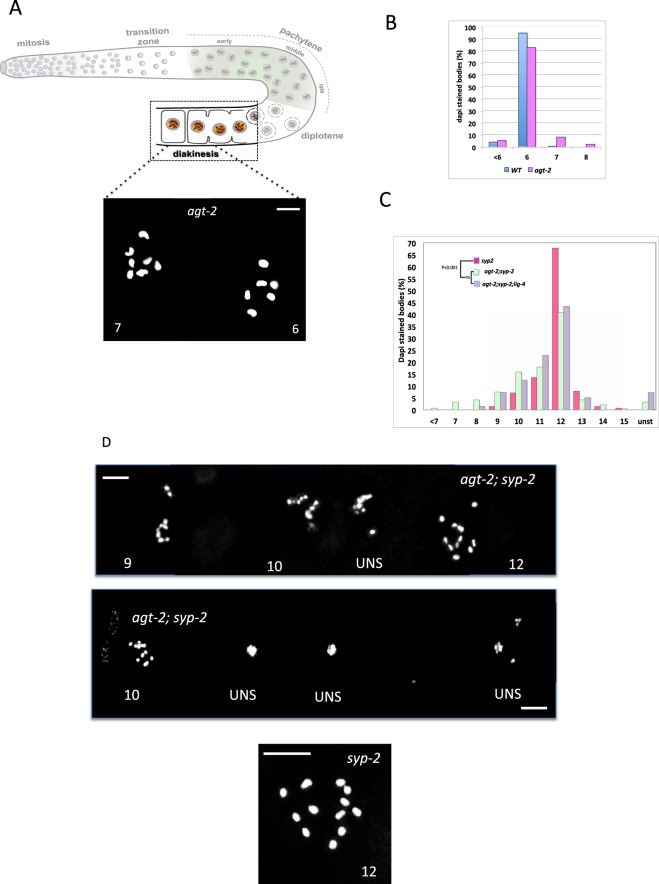
Analysis of DAPI stained bodies in diakinesis nuclei of the indicated genotypes. (**A**) Upper panel: Schematic depiction highlights the formation of bivalents (pairs of homologs) during dikinesis. Lower panel: Representative images of single DAPI stained oocyte nucleus at diakinesis from WT and *agt-2* mutant. The number of DAPI-stained bodies in each nucleus is shown. Scale bar: 2 μm. (**B**,**C**) Quantitation of DAPI-stained bodies. The y-axis in the histograms represents the percentage of nuclei in each class and the x-axis indicates the number of DAPI-stained bodies. Statistical analysis was carried out using one-way ANOVA multiple comparison test. A significance value of P < 0.05 was used; ns = not significant. (wt vs agt-2: p = 0.0015; syp-2 vs agt-2; syp-2: p < 0.001; agt-2; syp-2 vs agt-2; syp-2; lig.4: ns). Data are from five independent experiments. (**D**) Representative images of DAPI stained oocyte nuclei at diakinesis from indicated genotypes Scale bar: 2 μm. Nuclei for each genotype: N2 = 285, *agt-2* = 250, *syp-2* = 140, *agt-2; syp-2* = 240, *agt-2; syp-2; lig-4* = 100, *spo-11*: 100, *agt-2; spo-11*: 100.

Cytological analysis revealed that most of the diakinetic nuclei in *agt-2* mutant contain the wild-type complement of six bivalents at diakinesis; however, a significant number of *agt-2* nuclei had seven and, occasionally, eight DAPI stained bodies (Fig. 6A,B). The presence of more than six DAPI-stained bodies is usually observed in older wild type animals or in meiotic mutants in which proper segregation is inefficient due to lack of CO between two homologs. For instance, *brc-1* mutants are specifically defective in establishing a correct CO between the X chromosomes, which ultimately results in increased frequency of males^20,22^. Opposite to *brc-1*, *agt-2* has a normal frequency of males, while showing increased embryonic lethality and larval arrests (Table 1), which might result from missegregation of autosomes. This result might suggest that *agt-2* is largely dispensable for CO on the X chromosome, although it seems to contribute in establishing COs on autosomes.

In *syp-2* mutants meiotic DSBs occur normally; however, because of the absence of SC, which prevent homologous chromosome pairing, meiotic DSBs persist until late prophase, and can eventually be repaired using sister chromatids as template, maintaining chromosome integrity. Thus, *syp-2* meiocytes that reach diakinesis show 12 almost intact univalents^17^. Mutants with problems in the use of the sister chromatid as a template for meiotic DSB repair, such as *brc-1*, show higher number of univalent at diakinesis^20,22^.

We found that only about half of *agt-2; syp-2* diakinetic nuclei contain the expected 12 univalents, whereas a significant number of oocytes contained less then 12 DAPI stained bodies or unstructured, misshapen chromatin (Fig. 6C,D). These defects were not observed if meiotic DSBs were prevented: the expected complement of 12 univalents was found in the *agt-2*, *spo11* double mutant (Supplementary Fig. S4A,B), thus suggesting that the anomalies found in *agt-2*, *syp-2* diakinetic nuclei depend on meiotic DSBs and are due to inefficient repair of these lesions by using the sister chromatid as a template.

The reduced number of diakinetic chromosomes observed in the *agt-2; syp-2* mutants contrasts with chromosome fragmentation observed in *brc-1; syp-2* mutants, and is rather reminiscent of *fcd-2*; *syp-2* diakinetic oocytes^27^. These cells possess between 6 and 12 DAPI-stained bodies presumably due to inappropriate engagements between nonhomologous chromosomes during DNA repair, which were shown to be dependent on the alternative pathway for repair of DSBs, namely nonhomologous end joining (NHEJ). In order to establish whether NHEJ might play a role in the diakinetic abnormalities found in *agt-2*, we obtained the triple mutant *agt-2; syp-2; lig-4*, in which the NHEJ *lig-4* gene was mutated. No substantial rescue of the *agt-2* diakinetic defects was observed in the triple mutant (Fig. 6C). Thus, although *agt-2; syp-2* diakinetic oocytes do show problems in efficient use of sister chromatids to repair meiotic DSBs, this defect results neither in fragmentation, as observed in the absence of BRC-1, nor in NHEJ-dependent illegitimate repair between nonhomologous chromosomes, as in the absence of FCD-2. One possibility is that promiscuous non covalent chromosome aggregations occur in a fraction of *agt-2; syp-2* oocytes which are unable to perform correct sister-chromatid dependent homologues recombination (HR) to repair meiotc DSBs; these aggregations may result in reduction of the number of diakinetic chromosomes or, at most, massive chromosome destructuring (Fig. 6C,D).

In conclusion, the results reported in this paragraph indicate that *agt-2* is involved in both efficient CO on the autosomes and efficient inter-sister repair of meiotic DSBs.

### AGT-2 deficiency induces high levels of germ line apoptosis

Under normal growth conditions, about half of the oocytes in the *C*. *elegans* germ line undergo physiological cell death by the end of pachytene^40^. In addition, failure to faithfully execute meiosis triggers two checkpoints, called DNA damage and synapsis checkpoint, respectively^41–43^. Accumulation of DNA damage in the germ line, either in response to genotoxic stress, or upon failure to execute faithfully meiotic recombination triggers the DNA damage checkpoint, which result in cell cycle arrest at the G2 phase in order to allow for DNA repair. However, when level of DNA damage is excessive, cells might undergo apoptosis. Checkpoint-induced germ line apoptosis depends on the *C*. *elegans* p53 orthologue, CEP-1, removes defective meiotic nuclei to prevent aneuploidy and defective gametes and is restricted to late pachytene stage cells^44^.

**Figure 7:**
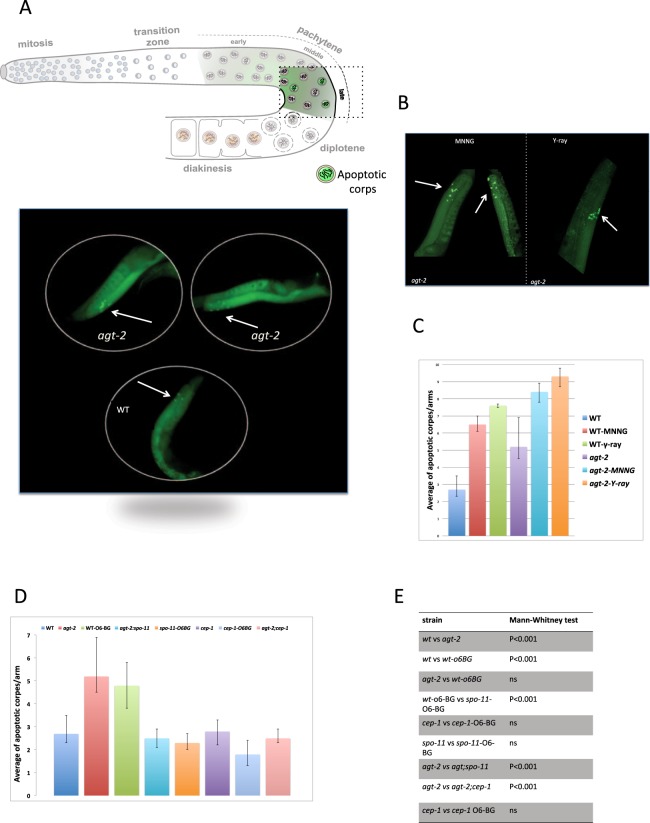
Analysis of apoptosis in the indicated genotypes or treatment. (**A**) Upper panel: germ cell apoptosis occurs mainly within the gonad loop region nearly as developing oocytes exit the pachytene stage of meiotic prophase (late pachytene). Schematic depiction shows the position of apoptotic corps highlighted in green. Lower panel: representative images showing apoptotic corps stained with SYTO-12, in the late pachytene zone of wild type and *agt-2* gonads. The arrows indicate the location of apoptotic nuclei. (**B**) Representative images showing apoptotic corps in the late pachytene zone of *agt-2* gonads treated with the indicated damaging agent; images were taken 16 hrs and 2 hrs after MNNG (10 μM) or γ-ray, respectively. (**C**,**D**) Quantification of apoptotic corps. Histograms show the average of apoptotic corps per gonad arm in the indicated genotypes or treatment. Error bars represent the standard error of the means (S.E.M.) calculated from at least three independent experiments. (**E**) Statistical comparisons between data sets were conducted using a two-tailed Mann-Whitney test. A significance value of P < 0.05 was used; ns = not significant. At least 80 gonads were scored for each condition.

Interestingly, we found that the average number of apoptotic pachytene nuclei was significantly higher in *agt-2* mutants than in the wild type during unperturbed growth (Fig. 7A,C and Supplementary Fig. 5). The number of apoptotic nuclei in *agt-2* animals was further increased by exposure to ionizing radiation or MNNG (Fig. 7B,C), indicating that the mutant strain is able to respond normally to DNA damage activating the DNA damage checkpoint (Fig. 7D,E).

We were therefore interested in determining whether the increased apoptosis observed in *agt-2* mutant was mediated by the *cep-1* pathway. For this, we generated double mutant strain *agt-2; cep-1* and then analyzed its phenotypes. We found that the embryonic lethality and brood size of the double mutant were similar to those observed in the *agt-2* mutant (brood size = 207 ± 6; embryonic lethality = 1.3% ± 0.2). In contrast to *agt-2* mutant, the increase in the number of apoptotic corps was not observed in *agt-2; cep-1* either under physiological conditions or after DNA damages induction. In addition, wild type worms treated with 2 mM O6-BG showed the same increase in the number of apoptotic nuclei found in untreated *agt-2* mutants (Fig. 7D,E and Supplementary Fig. 5). However, O6-BG did not induce apoptosis increase when *cep-1* was mutated (Fig. 7D,E). In order to clarify the nature of the damage triggering apoptosis elevation we analysed the apoptosis level in the absence of SPO-11: in *agt-2; spo-11* mutants and in *spo-11* mutants treated with O6-BG the level of apoptosis was similar to that found in untreated wild type animals (Fig. 7D,E).

Taken together, these results suggest the elevated apoptosis levels observed in the *agt-2* mutant or when the wild type was treated with the AGT inhibitor depend on inefficient repair of meiotic DSBs, which trigger the DNA damage checkpoint.

## Discussion

The predicted *C*. *elegans* AGT-2 protein has a peculiar primary structure, as compared with canonical AGT proteins; we here demonstrated that it also plays unexpected roles in the nematode biology. We have used different strategies to reduce or hamper AGT-2 activity: we analyzed an *agt-2* deletion strain, performed RNAi to down-regulate *agt-2* expression and used the AGT inhibitor O6-BG. In all circumstances we found the same range of relevant phenotypes: (i) brood size reduction; (ii) elevation of embryonic lethality and rate of developmental defects; (iii) accumulation and persistence of SPO-11 dependent RAD-51 foci; (iv) increased levels of SPO-11 and CEP-1 dependent germ line apoptosis. The increase in embryonic lethality and larval arrests, combined with the presence of more than six DAPI-stained bodies at diakinesis, suggests inefficient segregation of autosomes. In addition, we also evidenced that mutation of *agt-2* causes inefficient repair of meiotic DSBs using sister chromatids as template.

Collectively, these results suggest that AGT-2 is involved in efficient processing of meiotic DSBs, and that down-regulation or inactivation of the protein results in increased number of meiocytes that are incompatible with producing viable offspring and are eliminated by apoptosis.

Our results point to a role for AGT-2 in efficient conversion of HR intermediates into post-strand exchange products in meiosis. This result was unexpected, since the body of evidence available from experiments in other organisms suggests a specific role for AGT proteins in repair of alkylation damage. Experiments *in vitro*^29^ and evidence *in vivo* (this paper) suggest that AGT-2 is endowed with DNA-alkyl transferase activity. Indeed, *agt-2* mutant animals show increased sensitivity to alkylating agents and, most important, treatment of wild type animals with the AGT inhibitor O6-BG elicits the same phenotypes observed when the *agt-2* gene is mutated or down-regulated.

It is currently not clear whether the meiotic phenotypes observed are the consequence of the inactivation or impairment of the AGT-2 biochemical activity, and is difficult to envisage a role for such a specific activity in meiotic DSBs processing. One possibility is that AGT-2 plays a double role, i. e., it might catalyse direct repair of alkylating damage, thanks to its biochemical activity, and participate in the processing of meiotic DSBs by a still unclear mechanism. In this view, it is interesting to recall that several other *C*. *elegans* DNA repair genes have been implicated in meiotic recombination. These include, among others, *brc-1*, *fcd-2*, *msh-4* and *msh-5*^22,27,36,45^. The exact role of these genes in meiosis is not completely understood and is likely distinct. For instance, *brc-1* mutants show problems in X-chromosome segregation and inter-sister recombination^20,22^; *fcd-2* mutants compensate defects in HR by up-regulation of the NHEJ activity^27^; *msh-4* and *msh-5* mutants do not perform interhomolog COs and do not activate DNA-damage induced apoptotic response^45^. Notably, the range of the *agt-2* mutant phenotypes is not completely overlapping to that of any of the above mutants.

Data on the role of AGT proteins besides alkylation repair in other organisms are scarce. Knock out mice are viable and show no apparent defects, apart from higher sensitivity to alkylating agents-induced tumours^14,15^, although careful inspection of meiotic details in these animals has not been reported. On the other hand, several lines of evidence suggest that AGT proteins might participate in a number of important cellular functions. Proteomic analysis showed interaction of hAGT with a variety of cellular proteins^46^. Of particular interest is the interaction with proteins involved in DNA replication, recombination and repair (such as MCM2, PCNA, ORC1, DNA polymerase d, MSH-2, and DNA-dependent protein kinase), as well as histones. Notably, purified histone H1 and a combined histone fraction stimulate the AGT-catalyzed reaction *in vitro*^29^, an intriguing result in light of the peculiar presence, in AGT-2, of a histone-like domain, which raises the possibility that this domain has regulatory functions.

Intriguingly, in mouse embryonic fibroblasts and human cell lines, interaction of AGT with BRCA2 induced degradation of both proteins after treatment with alkylating agents or O6-BG^47^. Like BRCA2, the *C*. *elegans* hortholog BRC-2 facilitates nuclear localization of RAD-51 and its loading onto DNA^21^. This observation suggests a link between AGT-2 and the BRC-2-RAD-51 complex, which needs to be tested in further experiments.

Finally, AGT-2 might play a signaling role. This role is reminiscent of ATL proteins, that are very similar to AGTs, but catalytically inactive due a substitution of the active site cysteine. These proteins are believed to act as signal molecules retrieving other factors to damaged sites^48^. Thus, AGT-2 might function to integrate different DNA damage and repair signals, to ultimately ensure and maintain genomic stability.

## Supplementary information

Supplementary info
