# Supplementary material for: The DNA Alkylguanine DNA Alkyltransferase-2 (AGT-2) Of Caenorhabditis Elegans Is Involved In Meiosis And Early Development Under Physiological Conditions
Source: Sci Rep. 2019 May 3;9:6889. doi: 10.1038/s41598-019-43394-1 (PMC6499797; doi:10.1038/s41598-019-43394-1)
Supplement: Supplementary file 1 — Supplementary info [file 41598_2019_43394_MOESM1_ESM.pdf]

Supplementary Information

The DNA Alkylguanine DNA Alkyltransferase-2 (AGT-2) Of *Caenorhabditis Elegans* Is Involved In Meiosis And Early Development Under Physiological Conditions

Mario Serpe[§], Chiara Forenza[§], Adele Adamo, Noemi Russo, Giuseppe Perugino, Maria Ciaramella*[†] and Anna Valenti*

Table S1

strain	genotype	source
N2	*Wild type, Bristol*	CGC
/	*agt-2 (tm6462)II*	NBRP/ S. Mitani
AV106	*spo-11 (ok79)IV/[unc?(n754)let-?] (IV;V)*	CGC
VC172	*cep-1 (gk138) I*	CGC
RB873	*lig-4 (ok716) III*	CGC
AV276	*syp-2 (ok3017) V/nT1[unc-?(n754)let-? qls50](IV;V)*	CGC

Table S1: List of strains used in this study.

Table S2

gene	primers (5'-3')
agt-2	fwd: GTCACCCAGTAAGACAACC
	rev: TCCGAATTTTCTTGTTGTCGC
lig-4	fwd: AAAAAAAGTCGGCTCAAAAT
	rev: ACACCACTAACACAGACCAG
cep-1	fwd: TAAAATGGGATGTCTAGTGC
	rev: GAATGTCTTGGGATTAGAG
agt-2 (full gene)	fwd: CGTGAATCCGTCTATAAATACC
	rev: GCGACAACAAGAAAATTC

Table S2: Oligonucleotides used for PCR analysis of mutant genes.

Supplementary Information

The DNA Alkylguanine DNA Alkyltransferase-2 (AGT-2) Of *Caenorhabditis Elegans* Is Involved In Meiosis And Early Development Under Physiological Conditions

Mario Serpe[§], Chiara Forenza[§], Adele Adamo, Noemi Russo, Giuseppe Perugino, Maria Ciaramella*[†] and Anna Valenti*

Table S3

gene	RT-primers (5'-3')
agt-2	fwd: CGTGAATCCGTCTATAAATACC
	rev: GGTTGTCTTACTGGGTGAC
pmp-3	fwd: GTTCCCGTGTTCATCACTCAT
	rev: ACACCGTCGAGAAGCTGTAGA

Table S3: Oligonucleotides used for RT-PCR analysis. The oligonucleotides amplify about 200 bases from the exon 1 to exon 3 in *agt-2.*

Supplementary Information

The DNA Alkylguanine DNA Alkyltransferase-2 (AGT-2) Of *Caenorhabditis Elegans* Is Involved In Meiosis And Early Development Under Physiological Conditions

Mario Serpe[§], Chiara Forenza[§], Adele Adamo, Noemi Russo, Giuseppe Perugino, Maria Ciaramella*[†] and Anna Valenti*

Figure 1S

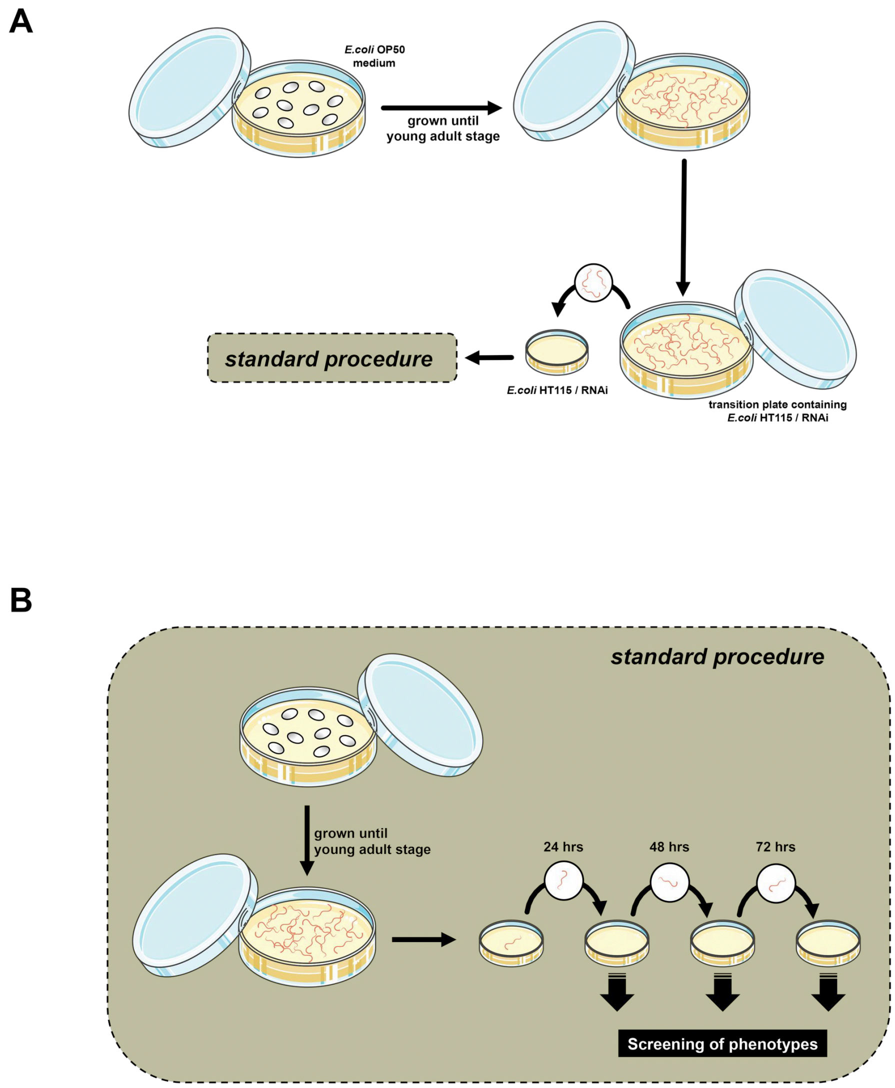

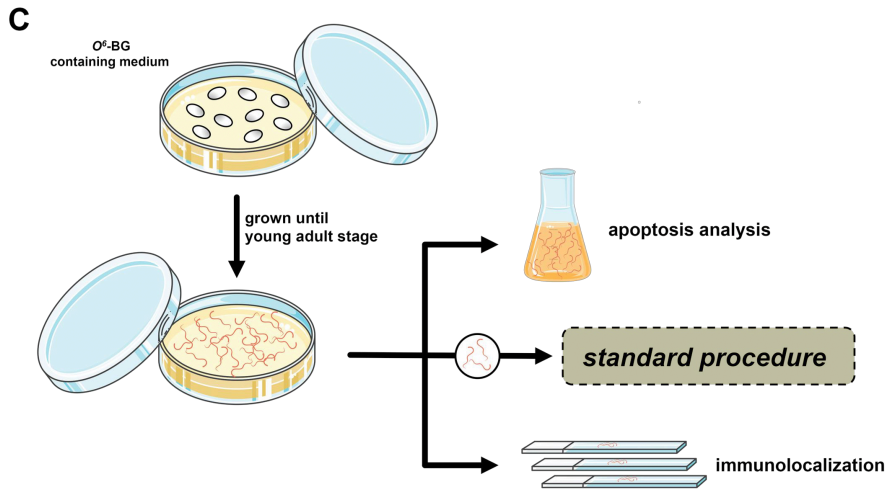

Figure 1S: Schematic representation of phenotypic screening experiments. (**A)** RNAi was performed by feeding wild type worms at L4 stage with bacteria transformed with the control pasmid L4440 or bacteria expressing dsRNA corresponding to either the *agt-1* or *agt-2* coding region. (**B)** Standard procedure used for phenotypic screening. (**C**) O6-BG treatment of worms and relative analysis.

Supplementary Information

The DNA Alkylguanine DNA Alkyltransferase-2 (AGT-2) Of *Caenorhabditis Elegans* Is Involved In Meiosis And Early Development Under Physiological Conditions

Mario Serpe§, Chiara Forenza§, Adele Adamo, Noemi Russo, Giuseppe Perugino, Maria Ciaramella*† and Anna Valenti*

Figure 2S

A

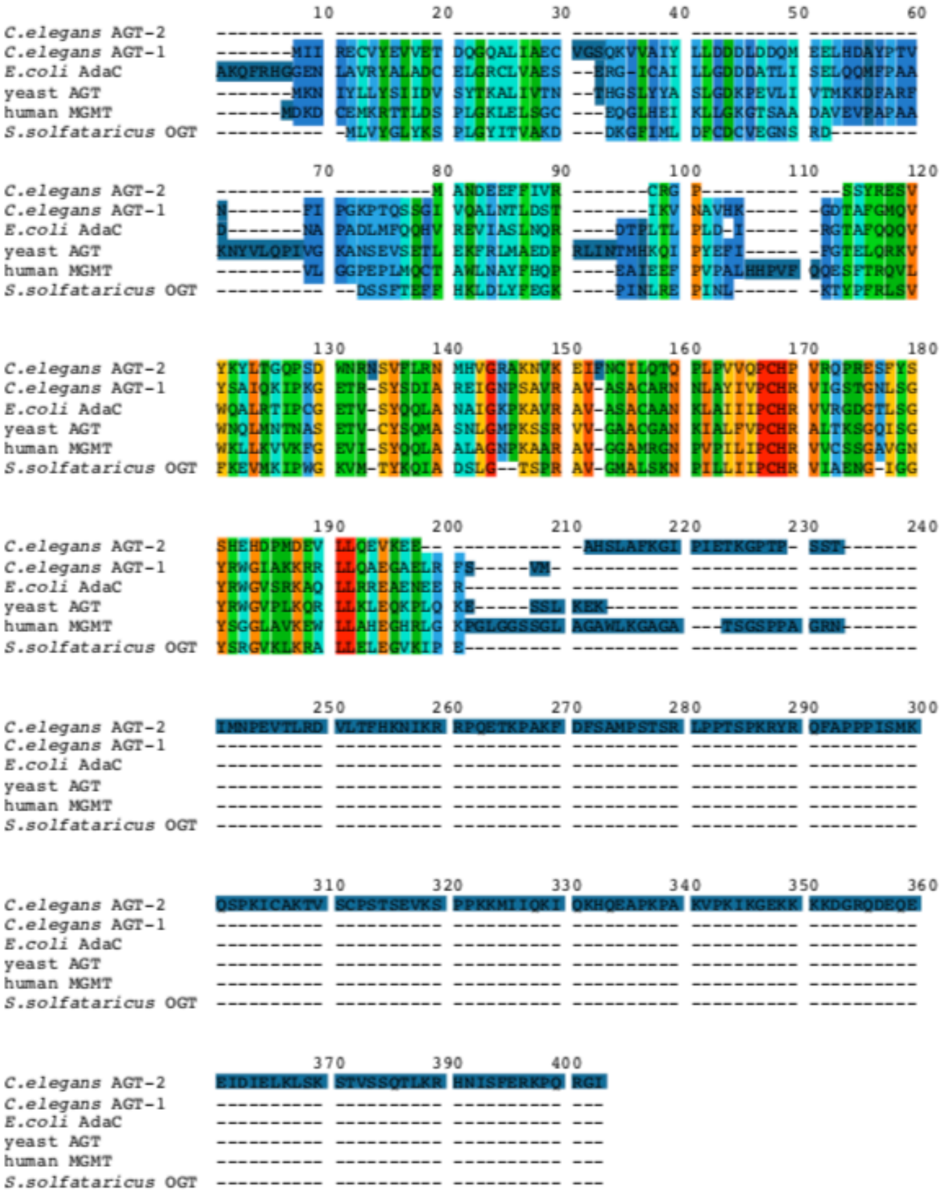

B

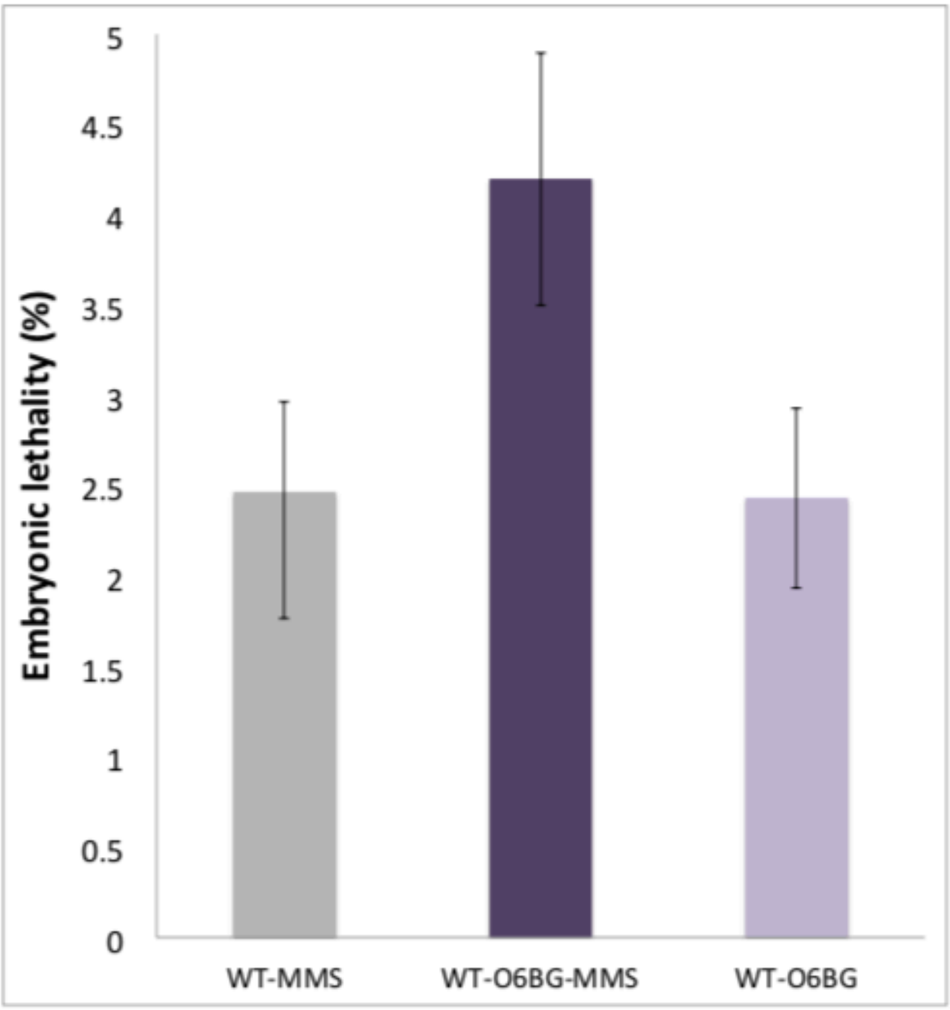

Figure 2S (A) Sequence alignment of selected AGTs. The alignment was obtained by the PRALINE program (http://www.ibi.vu.nl/programs/pralinewww/) and adjusted manually to fit the highly divergent AGT-2 sequence. Aminoacid conservation is indicated by color code. **(B)** Effect of alkylation damage on wild type worms pre-incubated with O6-BG and then treated with MMS as described in M & M section. Embryonic lethality was plotted as a percentage of the total unviable eggs on the total laid eggs. Error bars represent the standard error of the means (S.E.M.) calculated from three independent experiments.

Supplementary Information

The DNA Alkylguanine DNA Alkyltransferase-2 (AGT-2) Of *Caenorhabditis Elegans* Is Involved In Meiosis And Early Development Under Physiological Conditions

Mario Serpe[§], Chiara Forenza[§], Adele Adamo, Noemi Russo, Giuseppe Perugino, Maria Ciaramella*[†] and Anna Valenti*

Figure 3S

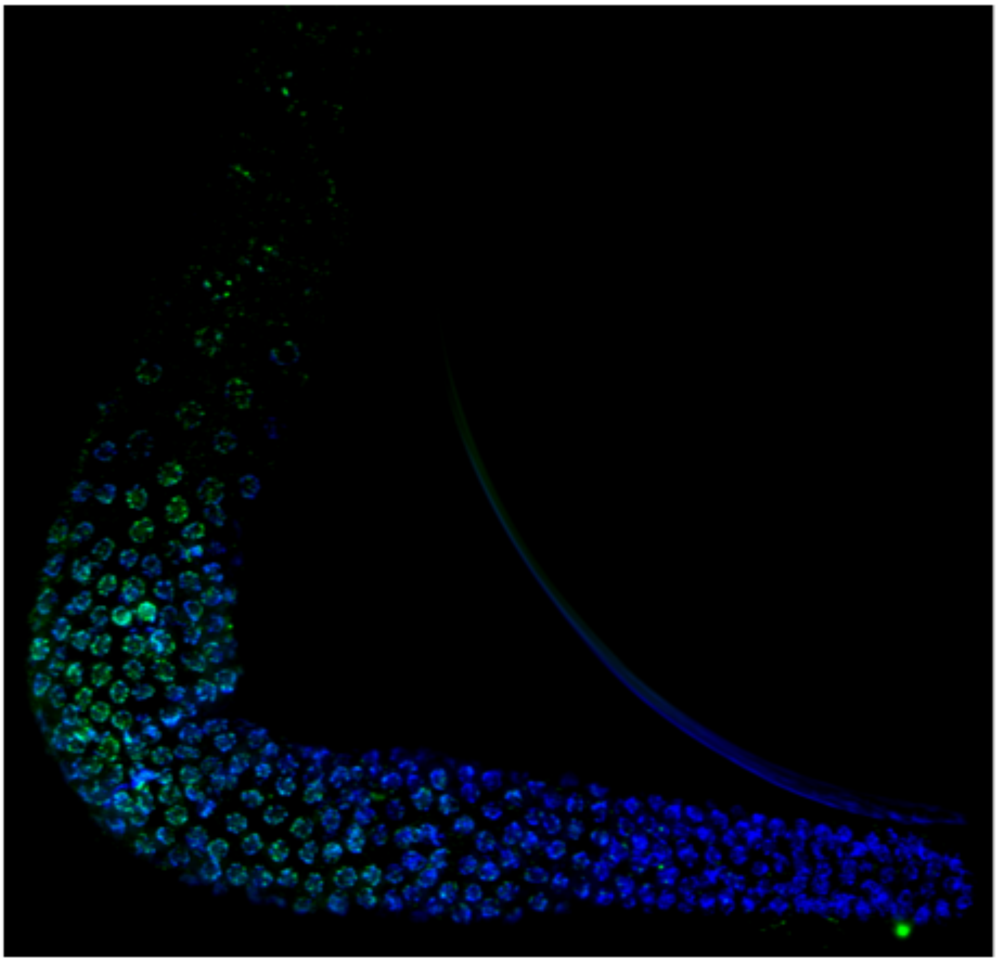

Figure 3S: Localization of synaptonemal complex. (**A**) Representative images of DAPI stained DNA (blue) and SYP-1 foci (green) detected with the anti-SYP-1 antibody in the gonad.

Supplementary Information

The DNA Alkylguanine DNA Alkyltransferase-2 (AGT-2) Of *Caenorhabditis Elegans* Is Involved In Meiosis And Early Development Under Physiological Conditions

Mario Serpe[§], Chiara Forenza[§], Adele Adamo, Noemi Russo, Giuseppe Perugino, Maria Ciaramella*[†] and Anna Valenti*

Figure 4S

A

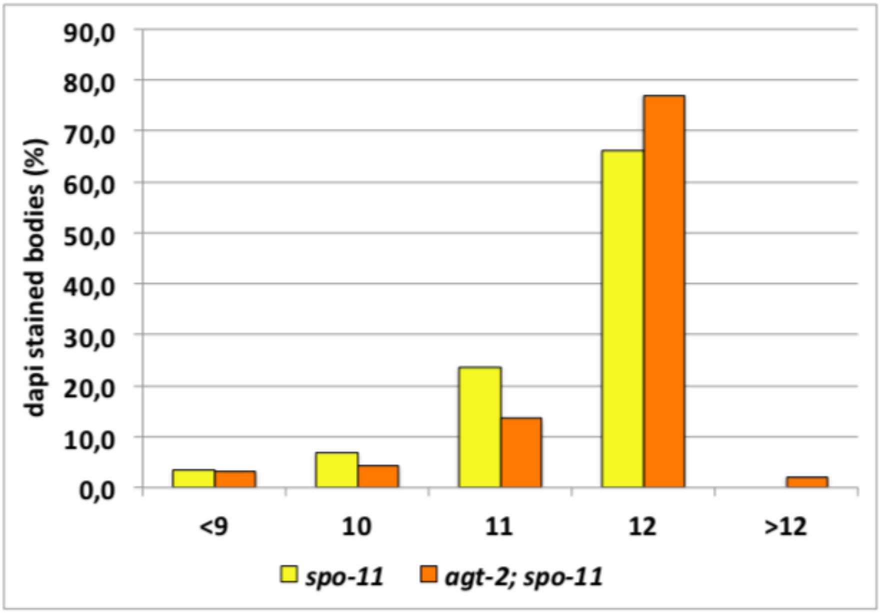

B

strain	Student t-test
spo-11 vs agt-2; spo-11	P=0.0863

Figure 4S: (A) Analysis of DAPI-stained bodies in diakinesis nuclei of *agt-2* and *agt-2;spo-11* mutants. The y-axis represents the percentage of nuclei in each class and the x-axis indicates the number of DAPI-stained bodies. **B**. Statistical analysis was carried out using the two-tailed paired Student's t tests. A significance value of $P < 0.05$ was used.

Supplementary Information

The DNA Alkylguanine DNA Alkyltransferase-2 (AGT-2) Of *Caenorhabditis Elegans* Is Involved In Meiosis And Early Development Under Physiological Conditions

Mario Serpe[§], Chiara Forenza[§], Adele Adamo, Noemi Russo, Giuseppe Perugino, Maria Ciaramella*[†] and Anna Valenti*

Figure 5S

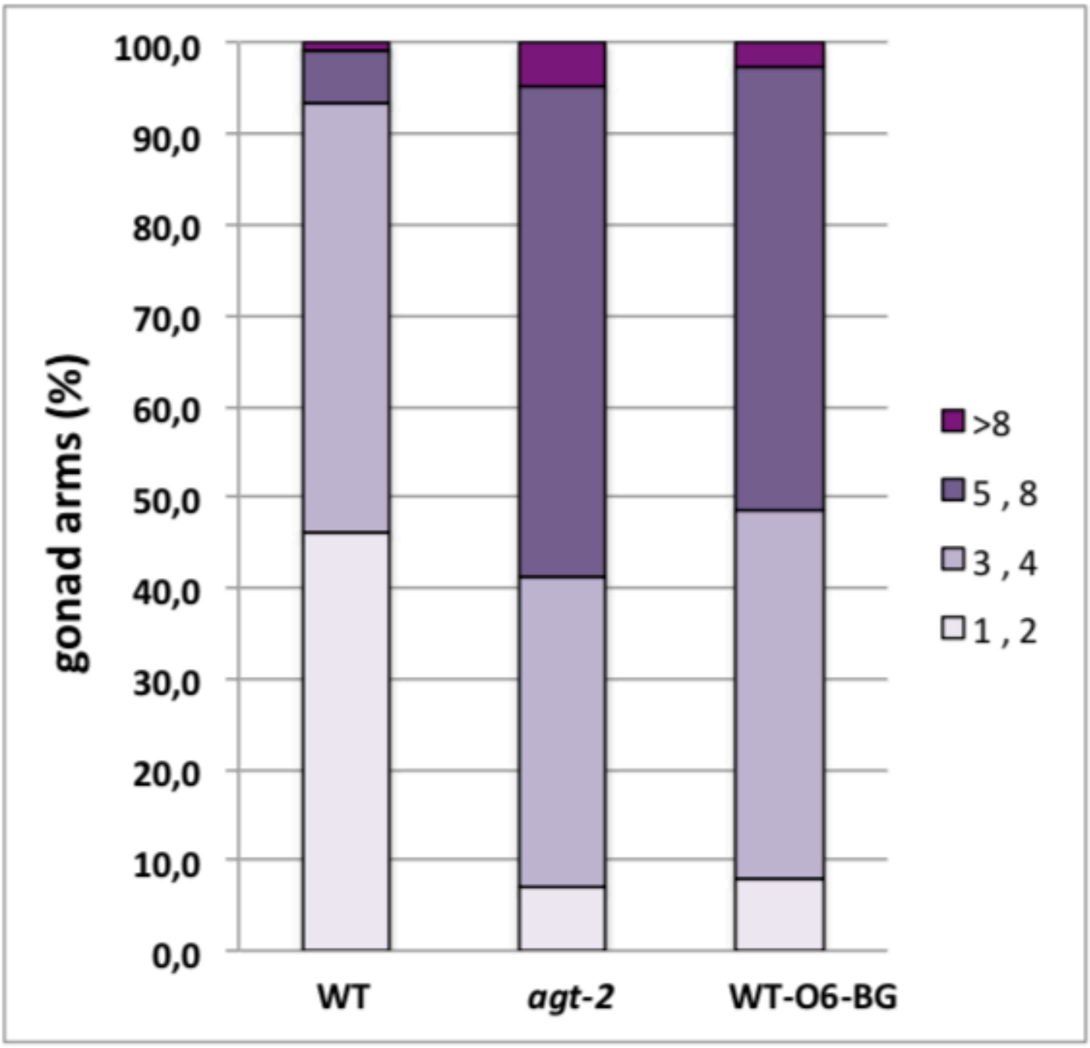

Figure 5S: Quantification of apoptosis in the indicated genotypes or after treatment with O6-BG (2 mM). Histogram shows the percentage of gonad arms with the indicated range of apoptotic corps.
